# Tumor cell-intrinsic PD-L1 promotes tumor-initiating cell generation and functions in melanoma and ovarian cancer

**DOI:** 10.1038/sigtrans.2016.30

**Published:** 2016-12-23

**Authors:** Harshita B Gupta, Curtis A Clark, Bin Yuan, Gangadhara Sareddy, Srilakshmi Pandeswara, Alvaro S Padron, Vincent Hurez, José Conejo-Garcia, Ratna Vadlamudi, Rong Li, Tyler J Curiel

**Affiliations:** 1 Department of Medicine, University of Texas Health Science Center, San Antonio, TX, USA; 2 The Graduate School of Biomedical Sciences, University of Texas Health Science Center, San Antonio, TX, USA; 3 Department of Molecular Medicine, University of Texas Health Science Center, San Antonio, TX, USA; 4 Department of Obstetrics and Gynecology, University of Texas Health Science Center, San Antonio, TX, USA; 5 Tumor Microenvironment and Metastasis Program, The Wistar Institute, Philadelphia, PA, USA; 6 Cancer Therapy and Research Center, University of Texas Health Science Center, San Antonio, TX, USA; 7 Department of Microbiology and Immunology, University of Texas Health Science Center, San Antonio, TX, USA

## Abstract

As tumor PD-L1 provides signals to anti-tumor PD-1^+^ T cells that blunt their functions, αPD-1 and αPD-L1 antibodies have been developed as anti-cancer immunotherapies based on interrupting this signaling axis. However, tumor cell-intrinsic PD-L1 signals also regulate immune-independent tumor cell proliferation and mTOR signals, among other important effects. Tumor-initiating cells (TICs) generate carcinomas, resist treatments and promote relapse. We show here that in murine B16 melanoma and ID8agg ovarian carcinoma cells, TICs express more PD-L1 versus non-TICs. Silencing PD-L1 in B16 and ID8agg cells by shRNA (‘PD-L1^lo^’) reduced TIC numbers, the canonical TIC genes *nanog* and *pou5f1* (*oct4*), and functions as assessed by tumorosphere development, immune-dependent and immune-independent tumorigenesis, and serial transplantability *in vivo*. Strikingly, tumor PD-L1 sensitized TIC to interferon-γ and rapamycin *in vitro.* Cell-intrinsic PD-L1 similarly drove functional TIC generation, canonical TIC gene expression and sensitivity to interferon-γ and rapamycin in human ES2 ovarian cancer cells. Thus, tumor-intrinsic PD-L1 signals promote TIC generation and virulence, possibly by promoting canonical TIC gene expression, suggesting that PD-L1 has novel signaling effects on cancer pathogenesis and treatment responses.

## Significance

Studies of PD-L1 signals in tumor immunopathogenesis and treatment responses have focused on cell-extrinsic effects. We introduce a major shift from the current paradigm by showing novel cell-intrinsic PD-L1 signaling that promotes TIC generation, virulence and treatment responses, greatly augmenting understanding of PD-L1 signals in cancer pathogenesis and treatment responses.

## Introduction

PD-L1 (CD274, B7-H1) is an immune co-signaling molecule in the B7-H (B7 homology) family. It has a key role in maintaining an immunosuppressive tumor environment by negatively regulating anti-tumor responses through fostering apoptosis, anergy or exhaustion of PD-1-expressing T cells, and is thus immunopathogenic in many cancers.^1,2^ Immune checkpoint blockade with anti-PD-L1 monoclonal antibodies (αPD-L1) is clinically effective in many cancer models. The αPD-L1 antibody atezolizumab was recently Food and Drug Administration approved to treat certain bladder and lung cancers. Its principal mode of action is thought to be protecting anti-tumor T cells from inhibition by tumor surface-expressed PD-L1.^1,2^ We recently reported that PD-L1 also mediates important cell-intrinsic signals that regulate immune-independent tumor growth, mTOR signaling and autophagy in melanoma and ovarian cancer cells.^3^ Tumor-intrinsic PD-L1 also regulates tumor glucose metabolism regulation, affecting anti-tumor T cells.^4^ Thus, cell-intrinsic PD-L1 signals merit further studies.

Tumors are comprised of genetically and functionally heterogeneous cell populations. Among these are tumor-initiating cells (TICs), initially reported in hematologic cancers, but that also give rise to epithelial carcinomas.^5,6^ Despite their importance, mechanisms for TIC generation and virulence are incompletely understood.

Because TICs are resistant to many therapies, and can give rise to tumor relapse, much attention has been given to targeting them for treatment.^5,6^ PD-L1 and PD-1 are expressed on TICs,^7–10^ but definitive evidence for their roles in TIC generation has not been reported to our knowledge. We demonstrate here that tumor cell-intrinsic PD-L1 directly drives the generation and functions of TICs in murine melanoma and ovarian cancer cells, and a human ovarian cancer cell line. Tumor PD-L1 is well known to give negative cell-extrinsic signals to PD-1^+^ T cells, but we now show that tumor PD-L1 also appears to generate intra-tumor cell signals that augment canonical TIC gene expression, regulate TIC numbers and functions, and sensitize them to rapamycin and interferon-γ. These previously unknown intracellular signaling effects define important new mechanisms for PD-L1 participation in cancer immunopathogenesis and suggests novel treatment strategies.

## Materials and methods

### Mice

Male and female wild-type (WT) C57BL/6J (BL6) and NOD.Cg-Prkdc^scid^Il2rg^tm1Wj1^/SzJ (non-obese diabetic/severe combined immunodeficiency (NOD/SCID)/interleukin-2Rγ KO, NSG) mice were purchased from Jackson Laboratory (Bar Harbor, ME, USA), maintained under specific pathogen-free conditions and given food and water *ad libitum*. Eight- to ten-week-old age- and sex-matched mice were used for experiments. All animal studies were approved by our Institutional Animal Care and Use Committee.

### Cell lines and transfections

The murine ovarian cancer cell line ID8, a gift from George Coukos, University of Pennsylvania, is a well-accepted transplantable mouse model that produces tumors replicating important aspects of human ovarian cancer, including local spread and ascites after intraperitoneal injection into syngeneic BL6 mice.^11^ We generated an aggressive ID8 line (ID8agg) by serial passage through WT mice.^3^ The murine melanoma B16-F10 cell line (herein ‘B16’) and the human ovarian cancer cell line ES2 were purchased from the American Type Culture Collection (Manassas, VA, USA). Cells were not re-authenticated for this work. Mouse cells are on the BL6 background. Cells were used in passages <5 and maintained in 5% fetal bovine serum-containing medium RPMI-1640 plus 1% penicillin/streptomycin, 1% l-glutamate and 1% HEPES buffer (complete medium). B16, ID8agg and human ES2 cells with stable PD-L1 knockdown were generated and described in our recent report.^3^ In brief, cells were transfected with murine or human control shRNA (‘control’) or shRNA against PD-L1 (‘PD-L1^lo^’). Monoclonal cells were selected in puromycin and PD-L1 expression status confirmed by flow cytometry and western blotting.

### *In vitro* conditions and treatments

All cells were cultured under identical conditions, including cell density and passage number, and studied at ~80% confluency on 100 mm plastic culture plates in complete medium. Indicated cultures included rapamycin (Sigma, St Louis, MO, USA) or mouse recombinant interferon-γ (R&D Systems, Minneapolis, MN, USA) for 48 h (B16 and ID8agg) or 60 h (ES2) at concentrations shown. Cell viability and numbers were determined on a Vi-Cell XR (Beckman Coulter, Brea, CA, USA) or hemocytometer and stained as described below. Dimethylsulfoxide (Sigma) or phosphate-buffered saline was used as negative controls (vehicle) for rapamycin or interferon-γ, respectively.

### Flow cytometry

Cells were stained and sorted as previously described,^12^ using BD LSRII and FACSAriaII hardware, and analyzed by FACSDiva (BD Bioscience, San Jose, CA, USA) or FlowJo (FlowJo LLC, Ashland, OR, USA) software. Zombie Yellow Fixable Viability kit, anti-mouse PD-L1 (B_v_421, clone 10F.9G2), CD44 (Per-CP-Cy5), CD133 (PE-Cy7), CD24 (PE), anti-human CD44 (Per-CP-Cy5), CD24 (PE), PD-L1 (PE-Cy7, clone 29E.2AE) and matched isotype controls were purchased from BioLegend (San Diego, CA, USA). ALDEFLUOR kits were purchased from STEMCELL Technologies (Vancouver, BC, Canada). The ALDEFLUOR assay was done with 1×10^6^ ES2 cells per ml. A volume of 5 μl of the specific aldehyde dehydrogenase (ALDH) inhibitor diethylaminobenzaldehyde was added to control tubes. Tubes were incubated with 5 μl BODIPY aminoacetaldehyde, a fluorescent substrate for ALDH and incubated for 45 min at 37 °C. Following washes, samples were kept at 4  °C for remaining staining. TICs were defined as CD44^+^CD133^+^CD24^+^ (B16)^13^, CD44^+^CD24^+^ (ID8)^14^ and ALDH^hi^ or CD44^+^CD24^−^ (as indicated, ES2)^15,16^ by flow cytometry.

### *In vivo* cell challenges, treatments and assessments

WT or NSG mice were injected with indicated numbers of B16 cells or corresponding TICs subcutaneously (s.c.),^17^ or ID8agg cells or corresponding TIC intraperitoneally B16 growth was measured with Vernier calipers and tumor volume calculated as (length×width^2^)/2. Survival was determined by tumor size ⩾1800 mm^3^ or animal distress. ID8agg survival was determined by ascites formation or distress.^18^ For serial re-transplantation, tumors were removed under sterile conditions when they reached 1800 mm^3^, digested, stained for viability and 10^4^ sorted TICs were injected into naive NSG mice subcutaneously. For ES2 TICs, female NSG mice were challenged with 20 000 sorted ALDH^hi^ TICs intraperitoneally. The survival of these mice was determined by ascites accumulation causing weight ⩾130% of baseline, or distress.

### Tumorosphere formation

10^4^ TICs were sorted from cultures and grown in DMEM-F12 medium (Gibco, Carlsbad, CA, USA) with B27 (Invitrogen, Carlsbad, CA, USA), 20 ng ml^−1^ epidermal growth factor (PeproTech, Rocky Hill, NJ, USA) and 20 ng ml^−1^ fibroblast growth factor (PeproTech)^14^ for 1–2 weeks in 25 cm^2^ flasks. At least six fields per clone were counted. Spheroid images were taken and analyzed using QCapture Pro 6.0 software (Surrey, BC, Canada). Diameter was calculated using the 100 μm measurement bar.

### Quantitative reverse transcription-PCR

Total RNA was isolated from cells using a Direct-zol RNA miniprep kit (Zymo Research, Irvine, CA, USA). Complementary DNA was synthesized with 1 μg total RNA using the ImPromII Reverse Transcription System (Promega, Madison, WI, USA) and random primers. Quantitative PCR was conducted using the 7900HT Real-Time PCR System (Applied Biosystems, Foster City, CA, USA), amplified with Taqman gene expression assays (Applied Biosystems) for mouse *nanog* (Mm02019550_s1), *sox2* (Mm03053810_s1), *pou5f1* (*oct4,* Mm03053917_g1) and *rptor* (Mm01242613_m1) according to the manufacturer’s instructions with *β-actin* (Mm02619580_g1) as the internal control. ES2 *NANOG* (Hs04399610_g1), *OCT4* (Hs00999632_g1), *SOX2* (Hs01053049_s1), *RPTOR* (Hs00375332_m1) and *GAPDH* were amplified in a similar manner. Fold changes were calculated by ΔΔCT between TICs and respective total cultures and *P*-value calculated by unpaired *t*-test. These fold changes were compared between control and PD-L1^lo^ cells by unpaired *t*-test.

### Statistical analysis

Statistical analyses were conducted using Prizm software (GraphPad, La Jolla, CA, USA). Data were plotted as means±s.e.m. For tumor growth, two-way analysis of variance plus Bonferroni post tests were used to compare replicate means. Unpaired *t*-test was used for comparison between individual means. Kaplan–Meier estimates and the log-rank test were used to analyze survival. *P*<0.05 was considered significant.

## Results

### TICs express higher PD-L1 and PD-1 versus non-TICs

**Figure 1:**
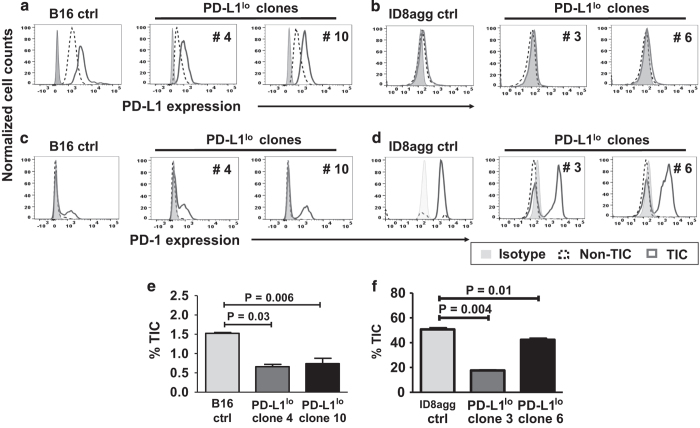
PD-L1 expression is higher in TICs versus non-TICs, and regulates TIC numbers *in vitro*. PD-L1 expression by flow cytometry in cultures of B16 (**a**) and ID8agg cells (**b**). PD-1 expression by flow cytometry in cultures of B16 (**c**) and ID8agg (**d**). TIC percentage for B16 (CD44^+^CD133^+^CD24^+^ cells), (**e**), ID8agg (CD44^+^CD24^+^ cells), (**f**) and respective PD-L1^lo^ clones by flow cytometry of cultured cells. *P*-values, unpaired *t*-test. Ctrl, control.

We investigated TIC PD-L1 expression on B16 and ID8agg cells by flow cytometry using well-accepted markers for each cell line^13,14^ (see Methods and Supplementary Figures 1 and 2). Both tumor lines expressed PD-L1, but interestingly, PD-L1 expression on B16 (Figure 1a) and ID8agg (Figure 1b) TICs were higher than respective non-TICs. Differential PD-L1 on TICs versus non-TICs was more pronounced in B16 (PD-L1 mean fluorescence intensity of control B16 TICs versus non-TICs, 4860 versus 1590; for PD-L1^lo^, clone 4, 1223 versus 589 and clone 10, 2075 versus 941; for control ID8agg, TICs versus non-TICs, 261 versus 215, PD-L1^lo^ clone 3, 132 versus 93 and clone 6, 209 versus 161 for TICs versus non-TICs, respectively). B16 and ID8agg PD-L1^lo^ TICs expressed lower PD-L1 versus their respective control TICs (Figures 1a and b) further validating the PD-L1 knockdown. B16 TICs express significant PD-1 consistent with a recent report,^19^ whereas B16 non-TICs express negligible PD-1. Similarly, ID8agg TICs expressed PD-1, whereas ID8agg non-TIC PD-1 expression was negligible. In both cell lines, PD-1 expression was similar in control versus PD-L1^lo^ TICs independent of PD-L1 expression (Figures 1c and d).

### Tumor PD-L1 regulates TIC numbers

We recently reported RNA-sequencing data from ID8agg cells, suggesting that cell-intrinsic PD-L1 controls canonical signaling pathways governing cell differentiation including mTOR. We also showed that PD-L1^lo^ B16 and ID8agg tumors grow slower versus respective control cells in immune-deficient mice.^3^ Thus, we hypothesized that PD-L1 signals could contribute to TIC generation as a mechanism for reduced growth kinetics and tumorigenicity. *In vitro* cultures of PD-L1^lo^ B16 cells had significantly fewer CD44^+^CD133^+^CD24^+^ TICs versus control B16 cultures (Figure 1e), and PD-L1^lo^ ID8agg cell cultures had significantly fewer CD44^+^CD24^+^ TICs versus PD-L1^lo^ ID8agg cell cultures (Figure 1f). Thus, tumor PD-L1 is a candidate regulator of TIC generation in melanoma and ovarian cancer cells.

### PD-L1 promotes TIC tumorosphere formation *in vitro*

**Figure 2:**
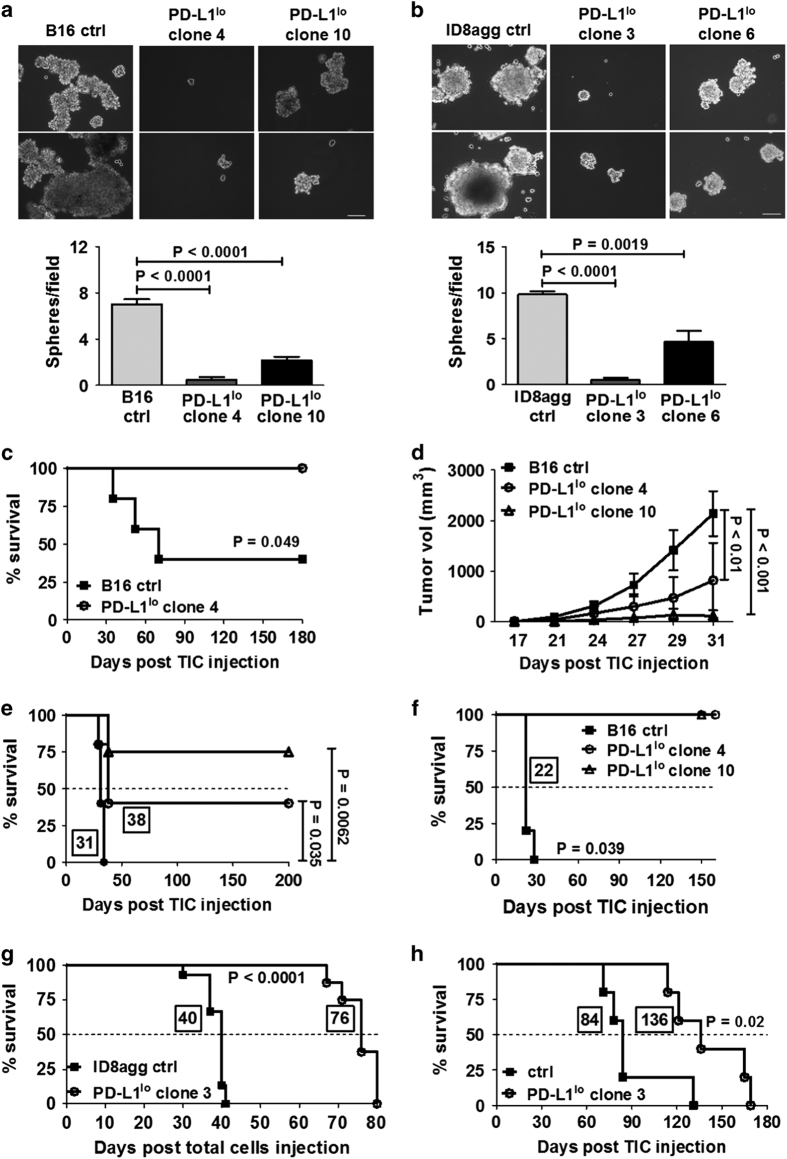
PD-L1^lo^ B16 and ID8agg TICs are functionally defective. Representative photomicrographs of tumorospheres after 14 days in culture of B16 (**a**) or ID8agg (**b**) control (ctrl) and PD-L1^lo^ clones. Bars equal 100 μm. Summary data below. At least six fields assessed per clone. *P-*values, unpaired *t*-test. (**c**) Survival of WT mice injected with 5000 indicated, sorted TICs (*n*=5–6 per group). *P*-value, log-rank test. (**d**) Tumor growth in NSG mice injected with indicated, sorted 10^4^ B16 TICs (*n*=5 per group). *P-*value, two-way analysis of variance. (**e**) Survival of mice in **d**. (**f**) % Survival in NSG mice injected with TICs sorted from tumors in **d**. *P*-value, log-rank test for trend. (**g**) Survival of WT mice injected intraperitoneal with 4×10^6^ total indicated ID8agg cells. (**h**) Survival of WT mice injected with 0.15×10^6^ ID8agg ctrl or PD-L1^lo^ clone 3 TIC. *P*-value, log-rank test. In **e**–**h**, boxed numbers indicate median survival (days).

A key functional aspect of TIC is self-renewal, studied *in vitro* as tumorosphere formation.^20^ PD-L1^lo^ TICs from B16 and ID8agg exhibited significantly reduced numbers and size of tumorospheres versus respective control TICs (Figures 2a and b), consistent with defective PD-L1^lo^ TIC self-renewal function *in vitro*.

### PD-L1 promotes immune-independent TIC growth and virulence *in vivo*

For *in vivo* functional assessment, control B16 TICs were challenged into WT mice, producing tumors with significantly shorter latency versus PD-L1^lo^ TICs (median 70 days versus >180 days (never reached, experiment terminated), respectively, Figure 2c). In fact, no mouse challenged with PD-L1^lo^ TICs formed tumors. Confirming tumorigenicity of control TIC, challenge with equal numbers of total cultured B16 cells did not produce detectable tumors in WT mice during this period (not shown). As PD-L1 can inhibit tumor rejection through immune mechanisms,^21^ we next challenged severely immune-deficient NSG mice with TICs from control or PD-L1^lo^ B16 TICs. Similar to the observations in WT mice, tumor growth was significantly slower (Figure 2d) and survival significantly longer (Figure 2e) in NSG mice following PD-L1^lo^ versus control B16 TIC challenge. Therefore, PD-L1 control of TIC growth and virulence includes cell autonomous and immune-independent mechanisms.

### TIC PD-L1 promotes TIC self-renewal *in vivo*

To confirm poor self-renewal potential of PD-L1^lo^ TICs, we assessed *in vivo* serial TIC passage.^20^ When tumors generated in NSG mice using TICs as above reached 1800 mm^3^, TICs were sorted from them and transplanted into naive NSG, producing control tumors with kinetics similar to the original transplant (compare Figure 2f with Figure 2e). By contrast, serial transplant of PD-L1^lo^ B16 TICs from similarly produced original PD-L1^lo^ tumors failed to produce tumors in NSG mice even after 150 days, when the experiment was terminated (Figure 2f). These data corroborate poor *in vitro* self-renewal of PD-L1^lo^ B16 TICs, confirm their defective *in vivo* function and demonstrate additional cell autonomous effects.

Total ID8agg control cells formed tumors significantly faster than PD-L1^lo^ ID8agg cells in WT mice (median survival 40 versus 76 days (*P*<0.0001), respectively, Figure 2g). Control ID8agg TICs were also significantly more tumorigenic than PD-L1^lo^ ID8agg TIC in WT mice (*P*=0.02, Figure 2h). These data confirm defective *in vivo* function in B16 and ID8agg PD-L1^lo^ TICs.

### PD-L1 promotes TIC genes

**Figure 3:**
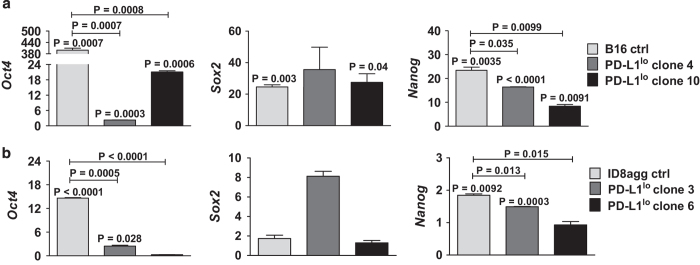
PD-L1 regulates canonical stemness genes. Fold change in gene expression (quantitative PCR) of *Oct4*, *Sox2* and *Nanog* in B16 (**a**) and ID8agg (**b**) cells. *P*-values, unpaired *t*-test. Each bar represents fold increase in stemness gene expression in TICs versus respective total cultures, with *P*-value for same directly over bar. *P-*values on horizontal lines compare fold changes between ctrl and PD-L1^lo^ cultures. Ctrl, control.

To understand mechanisms for PD-L1-mediated TIC effects, we hypothesized that PD-L1 promoted stemness gene expression. We thus assessed the stem cell master transcription factors *nanog*, *sox2* and *pou5f1* (*oct4*).^22^ In RNA-sequencing studies of total cultured ID8agg, these canonical TIC genes produced too few reads for statistics. Quantitative PCR analyses of highly purified, sorted B16 and ID8agg TICs demonstrated that *nanog* and *oct4*, but not *sox2* message were significantly higher in control or PD-L1^lo^ TICs versus their respective total cultures, and significantly lower in PD-L1^lo^ TICs versus respective control TICs (Figures 3a and b). In further support of PD-L1 control of stemness genes, the ovarian cancer TIC genes *cd24*, *cd117* (*c-Kit*) and *lin28a*^23^ and additional TIC genes *nes* and *ck18* were significantly reduced in total ID8agg PD-L1^lo^ versus control cells by RNA sequencing (Supplementary Table 1). Thus, PD-L1 regulates stemness gene expression, which is a candidate PD-L1-mediated TIC regulation mechanism.

### Cell-intrinsic PD-L1 promotes TIC interferon-γ sensitivity

**Figure 4:**
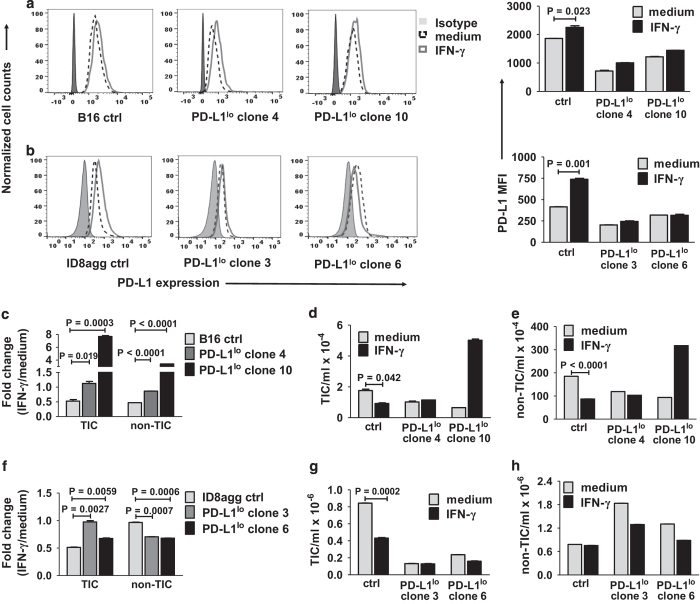
PD-L1 sensitizes B16 and ID8agg TICs to interferon-γ. Representative flow cytometry data for TIC PD-L1 expression on B16 (**a**) and ID8agg (**b**) cells treated with 0.2 ng ml^−1^ IFN-γ for 48 h, with summary PD-L1 mean fluorescence intensity (MFI) on the right. *P*-values, unpaired *t*-test. Fold change in B16 (**c**) TICs or non-TICs numbers after 0.2 ng ml^−1^ for 48 h, versus medium alone. Absolute numbers of B16 TICs (**d**) and non-TICs (**e**). Fold change in ID8agg (**f**) TICs and non-TICs treated with 0.5 ng ml^−1^ interferon-γ for 48 h, versus medium alone. *P*-values, unpaired *t*-test. Absolute numbers of ID8agg TICs (**g**) and non-TICs (**h**). Ctrl, control; IFN-γ, interferon-γ.

Interferon-γ is a potent PD-L1 inducer in tumor cell populations.^21^ B16 and ID8agg TICs expressed interferon-γ-inducible PD-L1, which expression was blunted in PD-L1^lo^ TIC as expected (Figures 4a and b), and also confirming the stringent PD-L1 knockdown effect in clones. We next tested interferon-γ-mediated growth suppression. Strikingly, control B16 TIC were more interferon-γ-susceptible versus corresponding PD-L1^lo^ TIC (Figure 4c). Further, the absolute numbers of control TICs and non-TICs were similarly reduced by interferon-γ treatment (Figures 4d and e), unlike PD-L1^lo^ TICs and non-TICs. Similarly, control ID8agg TICs were more susceptible to interferon-γ-mediated growth suppression versus control non-TICs, although it required an ~2.5-fold higher interferon-γ concentration to reduce ID8agg versus B16 TICs numbers (Figures 4f–h). PD-L1^lo^ ID8agg non-TICs were more sensitive versus control ID8agg non-TICs to interferon-γ-mediated growth suppression, whereas in B16, control and PD-L1^lo^ non-TICs were approximately equally sensitive (compare Figures 4c and f). These data suggest that interferon-γ signals in TICs can differ from non-TICs, that cell-intrinsic PD-L1 signals can affect interferon-γ effects in TICs and non-TICs, but there are also quantitative as well as qualitative tumor-specific differences that will require much additional work to clarify.

### Cell-intrinsic PD-L1 promotes TIC rapamycin sensitivity

**Figure 5:**
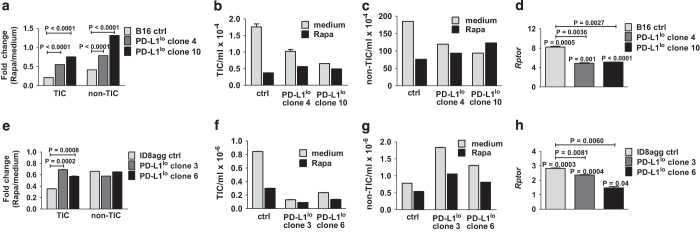
TIC PD-L1 sensitizes B16 and ID8agg to rapamycin. Fold change in numbers of B16 (**a**) TICs and non-TICs treated with 1.5 nm rapamycin for 48 h, versus medium alone. *P*-values, unpaired *t-*test. Absolute numbers of B16 TICs (**b**) and non-TICs (**c**). (**d**) Fold change in B16 *Rptor* expression by quantitative PCR (qPCR). Each bar represents fold increase in gene expression in TICs versus respective total cultures, with *P-*value for same directly over bar. *P*-values on horizontal lines compare fold changes between control (ctrl) and PD-L1^lo^ cultures. Fold change in numbers of ID8agg (**e**) TICs and non-TICs treated similarly with 1.5 nm rapamycin for 48 h, versus medium alone. Absolute numbers of ID8agg TICs (**f**) and non-TICs (**g**). (**h**) ID8agg *Rptor* expression by qPCR. *P-*values, unpaired *t*-test.

We reported that PD-L1 promotes mTORC1 signals in B16 and ID8agg cells.^3^ To test functional consequences in TICs, we cultured cells with rapamycin, which preferentially inhibits mTORC1.^24^ Growth of control B16 TIC was more rapamycin-sensitive versus corresponding control non-TICs, and in corresponding TICs, control TICs were more sensitive than PD-L1^lo^ TICs (Figures 5a–c). Thus, TICs can also differ in rapamycin-mediated growth suppression versus non-TICs and cell-intrinsic PD-L1 can control this effect in TICs as well as non-TICs. Regulatory associated protein of mTOR (*rptor*) is a positive mTOR regulator that acts as a scaffold for mTORC1 assembly and predicts mTORC1 activity. We used quantitative PCR of highly sort-purified TICs to show that *rptor* expression was significantly reduced in B16 PD-L1^lo^ TICs versus respective control TICs (Figure 5d), consistent with PD-L1-driven mTORC1 signaling in TICs as we reported for total cultures,^3^ and suggesting reduced mTORC1 dependence as an explanation for reduced rapamycin sensitivity in PD-L1^lo^ TICs. Similar to B16, control ID8agg TICs were more sensitive to rapamycin-mediated growth suppression versus PD-L1^lo^ TICs, but in contrast to B16, non-TICs were equally sensitive independent of PD-L1 status (compare Figures 5a–c with Figures 5e–g). *Rptor* was significantly reduced in ID8agg PD-L1^lo^ TICs (Figure 5h) consistent with PD-L1-driven augmentation of mTORC1 signaling, similar to mTORC1 augmentation in B16 TICs (Figure 5d).

### Tumor cell-intrinsic PD-L1 controls TIC numbers in human ovarian cancer cells

**Figure 6:**
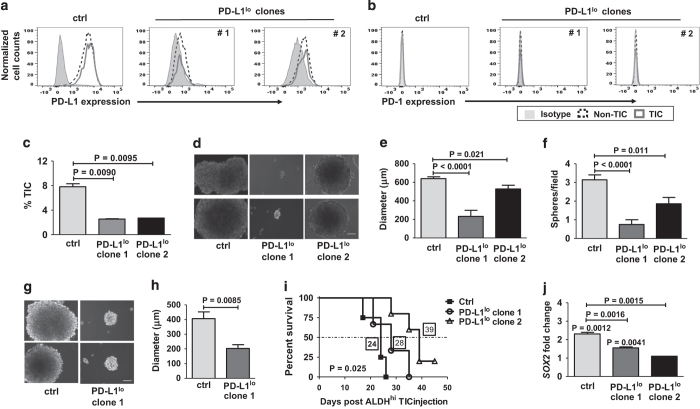
Human ES2 ovarian cancer TICs express PD-L1 that regulates self-renewal, immune-independent tumorigenicity and *SOX2*. PD-L1 (**a**) or PD-1 (**b**) expression in control (ctrl) and PD-L1^lo^ ES2 TIC by flow cytometry. (**c**) Percent of ALDH^hi^ TIC by flow cytometry of cultured cells. *P*-values, unpaired *t-*test. (**d**) Representative photomicrographs of ES2 tumorospheres. Bar equals 100 μm. Quantification of the size (**e**) and number of spheres per field (**f**). *P*-values, unpaired *t-*test. (**g**) Representative photomicrographs of tumorospheres formed by CD44^+^CD24^−^ TICs from control or PD-L1^lo^ clone 1 after 14 days, and their diameter (**h**). (**i**) Survival curve of NSG mice injected with 20 000 ALDH^hi^ TICs indicated (*n*=3–5 per group). Median survival (days) in boxes by individual curves. *P*-value, log-rank test. (**j**) *SOX2* expression by quantitative PCR from ALDH^hi^ TICs and total cultures. Fold changes by ΔΔCT between TICs and respective total cultures. These fold changes were compared between control and PD-L1^lo^ clones. All *P-*values calculated, unpaired *t-*test.

To assess human relevance of our data, we studied ES2 human ovarian cancer cells. We previously showed that these are PD-L1^+^, and that PD-L1^lo^ ES2 cells proliferated more slowly *in vitro* versus control ES2.^3^ ALDH1^hi^CD133^+^ is an accepted human ovarian cancer TIC phenotype.^15,16^ As we found that ES2 cells expressed negligible CD133 by flow cytometry (not shown), we used ALDH^hi^ expression to identify ES2 TICs *in vitro*. Control ES2 TICs expressed higher PD-L1 versus control non-TICs (PD-L1 mean fluorescence intensity 6300 versus 5437. In contrast to murine cells, PD-L1 expression in TICs was not different versus non-TICs PD-L1^lo^ clone 1, 912 versus 921 and clone 2, 1826 versus 1860 for TICs versus non-TICs, respectively; Figure 6a; Supplementary Figure 3). Further contrasting with mouse cell lines, PD-1 expression by flow cytometry was weak on control and PD-L1^lo^ TICs and non-TICs (Figure 6b). Nonetheless, cell-intrinsic PD-L1 controlled ES2 ovarian cancer TIC numbers *in vitro* (Figure 6c) as seen in mouse cells (Figures 1e and f).

### Cell-intrinsic PD-L1 controls tumorosphere formation and *SOX2* in human ES2 ovarian cancer cells

To assess TIC self-renewal function effects of PD-L1, we found that ES2 TIC cell-specific PD-L1 promoted tumorosphere formation *in vitro* as evidenced by significantly more numerous and bigger spheres than those formed by PD-L1^lo^ TIC (Figures 6d–f). As an alternative for ES2 TIC identification, CD44^+^CD24^−^ ES2 cells are also reported to have stem cell properties.^16^ We sorted CD44^+^CD24^−^ ES2 cells and showed that tumorosphere formation was also defective in PD-L1^lo^ versus control TICs defined this way (Figures 6g and h) consistent with the concept that cell-intrinsic PD-L1 in human ES2 cells controls self-renewal. Further, when we injected ALDH^hi^ TICs sorted from ES2 control or PD-L1^lo^ cultures into immunodeficient NSG mice, survival was significantly reduced in control versus PD-L1^lo^ TIC challenge (Figure 6i), consistent with reduced TIC tumorigenicity function of PD-L1^lo^ ES2 TICs *in vivo*. ES2 TICs overexpress the canonical stem cell genes *SOX2* and *OCT4*.^16^ We sorted ALDH^hi^ ES2 TICs and used quantitative PCR to show that expression of *SOX2* (Figure 6j), but not *OCT4* or *NANOG* (Supplementary Figure 4), was significantly reduced in PD-L1^lo^ ES2 TICs versus control TICs, but *SOX2* in control or PD-L1^lo^ TICs was higher than in respective total cultures. Thus, PD-L1 in human ES2 ovarian cancer cells recapitulates important TIC features and functions seen in the murine cell lines, although PD-L1 influences different stemness genes in these distinct cellular contexts.

### Tumor-intrinsic PD-L1 regulates interferon-γ and rapamycin responsiveness in human ES2 ovarian cancer cells

**Figure 7:**
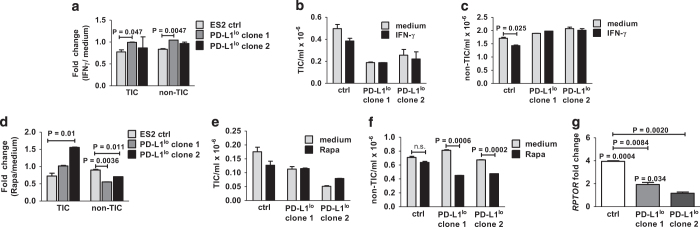
Human ES2 ovarian cancer TIC PD-L1 alters rapamycin (rapa) and interferon (IFN)-γ sensitivity. (**a**) Fold change in numbers of ES2 TICs and non-TICs treated with 0.5 ng ml^−1^ IFN-γ for 60 h, versus medium alone. *P*-values, unpaired *t*-test. Absolute numbers of TICs (**b**) and non-TICs (**c**). Fold change in numbers of ES2 TICs (**d**) and non-TICs treated similarly with 5 nm rapamycin for 60 h, versus medium alone. Absolute numbers of TICs (**e**) and non-TICs (**f**). (**g**) Fold change in *RPTOR* expression by quantitative PCR. Each bar represents fold increase in gene expression in TICs versus respective total cultures, with *P*-value for same directly over bar. *P*-values on horizontal lines compare fold changes between ctrl and PD-L1^lo^ cultures. All *P-*values, unpaired *t*-test.

Finally, we investigated the sensitivity of ES2 TIC to growth inhibition by interferon-γ and rapamycin. Control ES2 TICs and non-TICs were more sensitive than respective PD-L1^lo^ TICs and non-TICs to growth suppression by interferon-γ (Figures 7a–c). PD-L1 similarly rendered ES2 TICs sensitive to rapamycin-mediated growth inhibition. By contrast to ES2 TIC effects, PD-L1 inhibited non-TIC rapamycin-mediated growth inhibition (Figures 7d–f). mTORC1 signals were reduced comparably to PD-L1^lo^ B16 and ID8agg as assessed by *RPTOR* gene expression levels (compare Figure 7g with Figures 5d and h).

## Discussion

Mechanisms regulating TIC generation, proliferation and virulence remain incompletely understood. As the initial observation that tumor cell-expressed PD-L1 (B7-H1) can kill T cells,^1^ the focus of PD-L1 effects on tumor immunopathology has been on cell-extrinsic inhibitory signals to PD-1^+^ T cells.^1,2,17,25^ We recently reported that tumor PD-L1 regulates cell autonomous immune-independent tumor growth, mTOR and autophagy in melanoma and ovarian cancer cells.^3^ In this study, we show additional significant, previously unrecognized effects of tumor-intrinsic PD-L1 on TIC generation, proliferation, canonical TIC gene expression and treatment responses differing from known tumor extrinsic PD-L1 effects, and demonstrate their human relevance.

PD-L1 is expressed by cancer stem cells in human glioma,^7^ squamous cell carcinoma of the head and neck,^9^ and colon cancer where PD-L1 was higher than non-stem cells,^8^ but a mechanistic relationship between PD-L1 and stem cell generation or function was not defined. We show here that TICs express higher PD-L1 levels than non-TICs in mouse melanoma and mouse but not in human ovarian cancer. This cell-intrinsic PD-L1 controlled numbers of TIC generated *in vitro* in mouse B16 melanoma and ID8agg ovarian cancer cells, and in human ES2 ovarian cancer cells. Thus, this novel capacity of cell-intrinsic PD-L1 to promote TICs is not confined to a single tumor type and is also relevant to human cancer. The significance of higher PD-L1 expression in some TICs versus non-TICs is uncertain, but could relate to additional TIC-specific signaling in differing tumor cell types. In human head and neck cancers, cancer stem cells defined solely by CD44 expression and sorted into PD-L1^+^ and PD-L1^−^ populations effected comparable tumorigenicity in NSG mice, whereas PD-L1^+^ stem cells elicited more evidence for T-cell dysfunction *in vitro* versus PD-L1^−^ stem cells.^9^ By contrast, we show here that PD-L1^lo^ TICs in melanoma and ovarian cancer had poor tumorigenicity in immune sufficient and immune-deficient mice, thus confirming that TIC-intrinsic PD-L1 has unexpected TIC growth promoting effects in melanoma and ovarian cancer cells, in addition to anticipated immune evasive effects. PD-L1 mediates an immune evasive role in all cancers and TIC tested thus far. The lack of clear PD-L1-mediated immune-independent TIC growth promotion in head and neck cancers that contrasts with our data in melanoma and ovarian cancer cells suggests that cell-intrinsic PD-L1 growth promotion also depends on tumor-specific properties yet to be defined, an area worthy of additional study. The above mentioned study tested fewer mice and assessed one dose of TICs, which could help explain the divergent outcomes.

We recently showed that in total B16 and ID8agg cell cultures, tumor PD-L1 promoted tumor cell proliferation and mTORC1 activation.^3^ We show here that PD-L1 increased the mTORC1 component *rptor* in B16 and ID8agg TIC, and also in human ES2 TIC, consistent with increased mTORC1 signals. The preferential mTORC1 inhibitor rapamycin reduced control (PD-L1 replete) B16, ID8agg TIC numbers *in vitro* with significantly diminished effects on PD-L1^lo^ TIC, consistent with the concept that PD-L1-driven mTORC1 promotes TIC generation in these cells. TIC PD-L1 also sensitized human ES2. TICs are considered to be treatment resistant, but we show that TIC PD-L1 sensitizes TICs to the small-molecule mTORC1 inhibitor rapamycin. This observation deserves additional exploration, including assessing other mTORC1 or dual mTORC1/2 inhibitors. Our data predict that efficacy of αPD-L1 in PD-L1^+^ tumors for which αPD-L1 is most likely to be effective^26,27^ could be enhanced in some cases when combined with an appropriate mTOR inhibitor.

Interferon-γ-induced PD-L1 regulation is well known.^27^ Thus, as expected, interferon-γ augmented TIC PD-L1 expression. However, we show here that PD-L1 also appears to alter interferon-γ signals, as TIC PD-L1 unexpectedly sensitized murine B16 and ID8agg TICs, and human ES2 TICs, to interferon-γ-mediated growth suppression *in vitro*. These data demonstrate the first TIC immune-sensitizing mechanism to our knowledge. In adaptive immune resistance, the incoming immune response, including interferon-γ, makes the tumor resist further immune attack.^28^ TIC PD-L1 might suppress anti-tumor T-cell responses as seen in studies of total tumors^1,21^ and head and neck cancer TIC,^9^ yet TIC PD-L1 could render them more vulnerable to immune-mediated clearance. This complexity requires much additional study to understand in which TIC their PD-L1 alters immune effects on tumor and to optimize immunotherapy treatment strategies. For example, our work suggests that αPD-L1 plus interferon-γ, T cells producing interferon-γ (such as TIC-targeting CAR T cells) or perhaps interferon-α are strategies for further testing in TICs in which PD-L1 sensitizes them to interferon-γ-mediated growth inhibition.

In assessing mechanisms for TIC generation, we found that PD-L1 signals promoted canonical stemness gene expression. In mouse B16 melanoma and ID8agg ovarian cancer cells, PD-L1 augmented expression of *oct4* and *nanog*, but not *sox2.* By contrast, PD-L1 promoted *SOX2* but not *OCT4* or *NANOG* in human ES2 ovarian cancer cells. These data are consistent with the notion that PD-L1-driven stemness gene expression is a mechanism for its capacity to generate TIC. Our data further suggest that the specific stemness genes driving TIC depend on the tumor, likely reflecting differing mutational landscapes in ovarian cancers versus melanomas,^29,30^ and also helping understand the many cell-specific outcomes, we define here in regards also to rapamycin and interferon-γ sensitivity. Mechanisms for how PD-L1 alters gene expression requires further study. Effects are transcriptional, as evidenced by reduced canonical stem cell gene message in PD-L1^lo^ TICs we show here and in our recent work on non-TICs;^3^ however, how PD-L1 effects transcriptional control remains to be defined. Regulation could also be through mTOR-related translational effects.^31^ Understanding these specific mechanisms could define novel means to reduce TIC generation.

PD-1, a major PD-L1 receptor, was recently shown to regulate cell-intrinsic and immune-independent melanoma growth in mouse and human melanoma cells.^10^ B7-H3, such as PD-1 and PD-L1, is an immunoglobulin superfamily member. These three molecules all exert tumor cell-intrinsic effects, including regulating tumor mTOR, its regulators or its targets.^3,10,32^ As mTOR could have a critical role in TIC generation,^33^ the concept that tumor cell-intrinsic immunoglobulin superfamily members exert related, important signaling effects in cancer immunopathogenesis or treatment responses merits much additional attention.

In summary, we show that tumor PD-L1 effects are considerably broader than simply blunting PD-1^+^ anti-tumor T-cell activities and suggest many additional avenues for investigations of tumor immunopathogenesis, treatment approaches and potentially treatment responses. Given the virulence and treatment resistance of TIC, such studies could also help develop successful new treatment approaches that specifically target TICs. Finally, immune checkpoint molecules in the immunoglobulin superfamily could have common tumor cell-intrinsic properties that will expand our understanding of their contributions to cancer pathogenesis and provide insights into effective cancer treatments.
