# Supplementary material for: Tumor cell-intrinsic PD-L1 promotes tumor-initiating cell generation and functions in melanoma and ovarian cancer
Source: Signal Transduct Target Ther. 2016 Dec 23;1:16030–. doi: 10.1038/sigtrans.2016.30 (PMC5547561; doi:10.1038/sigtrans.2016.30)
Supplement: Supplementary Table 1 [file sigtrans201630-s1.pdf]

Suppl. Table 1

Gene	Fold change (PD-L1^{lo}/ctrl)	P value
cd24	**0.0670**	**1.39E-11**
c-kit	**0.0158**	**3.03E-39**
lin28a	**0.1235**	**4.62E-18**
nes	**0.3096**	**3.47E-15**
ck18	**0.6185**	**1.10E-03**

RNA-Seq data from the ID8agg and ID8agg PD-L1^{lo} clone 3 total cells for ovarian cancer associated stemness genes. Fold changes were calculated as the ratio of expression in PD-L1^{lo} cells compared to control cells.
