# Supplementary material for: Tumor cell-intrinsic PD-L1 promotes tumor-initiating cell generation and functions in melanoma and ovarian cancer
Source: Signal Transduct Target Ther. 2016 Dec 23;1:16030–. doi: 10.1038/sigtrans.2016.30 (PMC5547561; doi:10.1038/sigtrans.2016.30)
Supplement: Supplementary Figure 1 [file sigtrans201630-s2.ppt]

## Slide 1
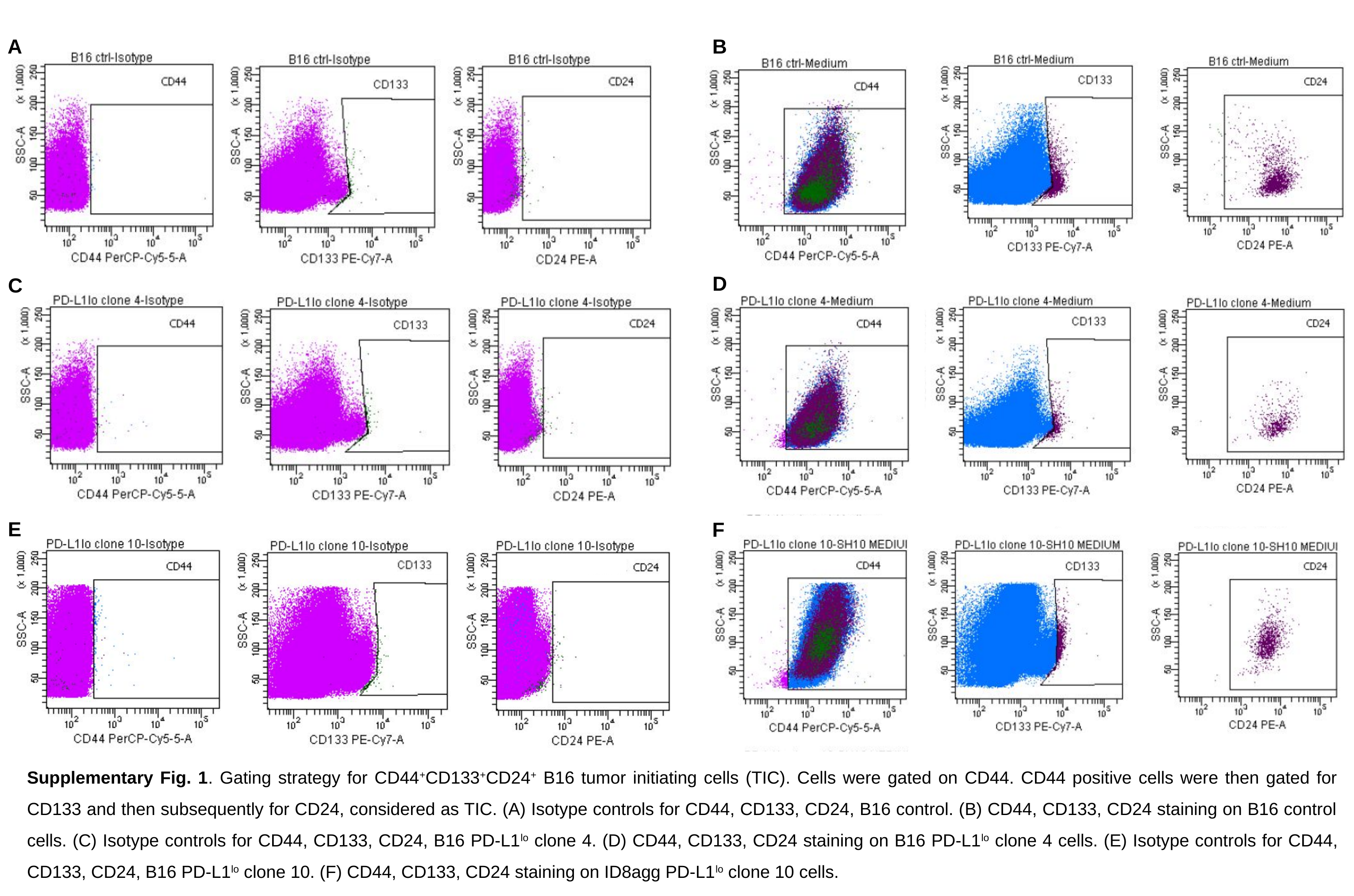

A
B
D
C
E
F
Supplementary Fig. 1. Gating strategy for CD44+CD133+CD24+ B16 tumor initiating cells (TIC). Cells were gated on CD44. CD44 positive cells were then gated for CD133 and then subsequently for CD24, considered as TIC. (A) Isotype controls for CD44, CD133, CD24, B16 control. (B) CD44, CD133, CD24 staining on B16 control cells. (C) Isotype controls for CD44, CD133, CD24, B16 PD-L1lo clone 4. (D) CD44, CD133, CD24 staining on B16 PD-L1lo clone 4 cells. (E) Isotype controls for CD44, CD133, CD24, B16 PD-L1lo clone 10. (F) CD44, CD133, CD24 staining on ID8agg PD-L1lo clone 10 cells.
