# Supplementary material for: Tumor cell-intrinsic PD-L1 promotes tumor-initiating cell generation and functions in melanoma and ovarian cancer
Source: Signal Transduct Target Ther. 2016 Dec 23;1:16030–. doi: 10.1038/sigtrans.2016.30 (PMC5547561; doi:10.1038/sigtrans.2016.30)
Supplement: Supplementary Figure 2 [file sigtrans201630-s3.ppt]

## Slide 1
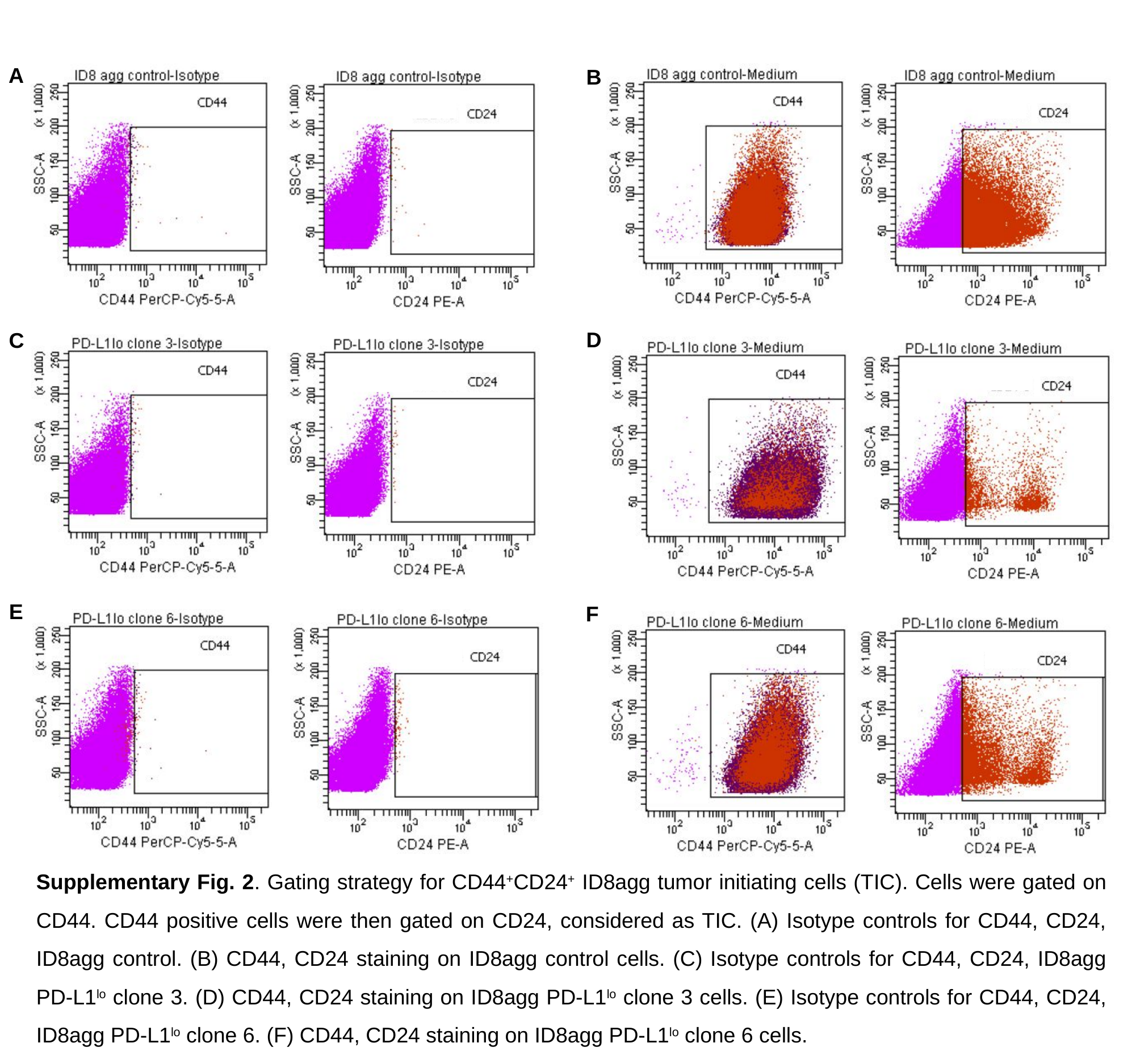

A
B
D
C
E
F
Supplementary Fig. 2. Gating strategy for CD44+CD24+ ID8agg tumor initiating cells (TIC). Cells were gated on CD44. CD44 positive cells were then gated on CD24, considered as TIC. (A) Isotype controls for CD44, CD24, ID8agg control. (B) CD44, CD24 staining on ID8agg control cells. (C) Isotype controls for CD44, CD24, ID8agg PD-L1lo clone 3. (D) CD44, CD24 staining on ID8agg PD-L1lo clone 3 cells. (E) Isotype controls for CD44, CD24, ID8agg PD-L1lo clone 6. (F) CD44, CD24 staining on ID8agg PD-L1lo clone 6 cells.
