# Supplementary material for: Tumor cell-intrinsic PD-L1 promotes tumor-initiating cell generation and functions in melanoma and ovarian cancer
Source: Signal Transduct Target Ther. 2016 Dec 23;1:16030–. doi: 10.1038/sigtrans.2016.30 (PMC5547561; doi:10.1038/sigtrans.2016.30)
Supplement: Supplementary Figure 3 [file sigtrans201630-s4.ppt]

## Slide 1
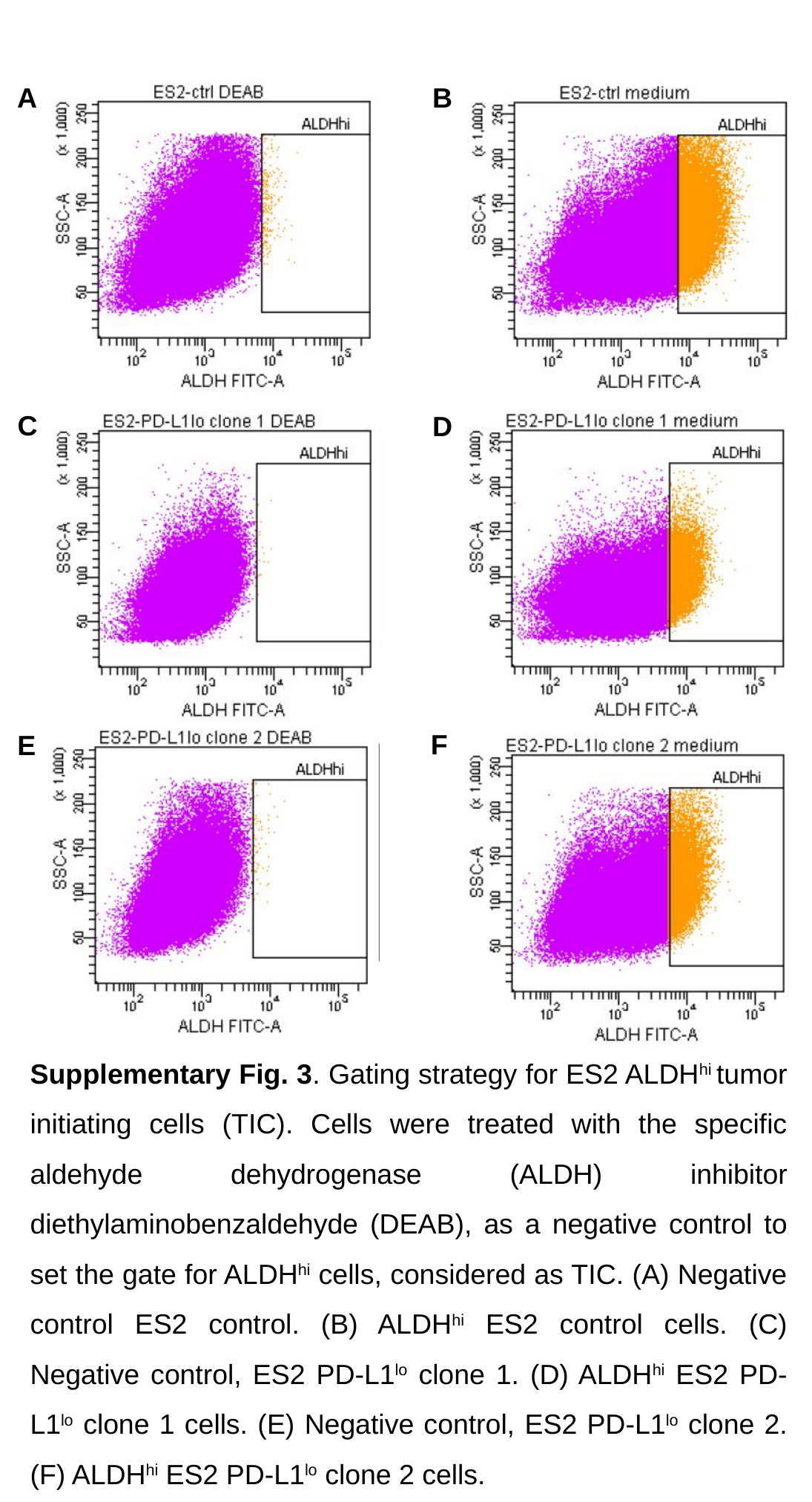

A
B
C
D
F
E
Supplementary Fig. 3. Gating strategy for ES2 ALDHhi tumor initiating cells (TIC). Cells were treated with the specific aldehyde dehydrogenase (ALDH) inhibitor diethylaminobenzaldehyde (DEAB), as a negative control to set the gate for ALDHhi cells, considered as TIC. (A) Negative control ES2 control. (B) ALDHhi ES2 control cells. (C) Negative control, ES2 PD-L1lo clone 1. (D) ALDHhi ES2 PD-L1lo clone 1 cells. (E) Negative control, ES2 PD-L1lo clone 2. (F) ALDHhi ES2 PD-L1lo clone 2 cells.
