# Supplementary material for: Tumor cell-intrinsic PD-L1 promotes tumor-initiating cell generation and functions in melanoma and ovarian cancer
Source: Signal Transduct Target Ther. 2016 Dec 23;1:16030–. doi: 10.1038/sigtrans.2016.30 (PMC5547561; doi:10.1038/sigtrans.2016.30)
Supplement: Supplementary Figure 4 [file sigtrans201630-s5.ppt]

## Slide 1
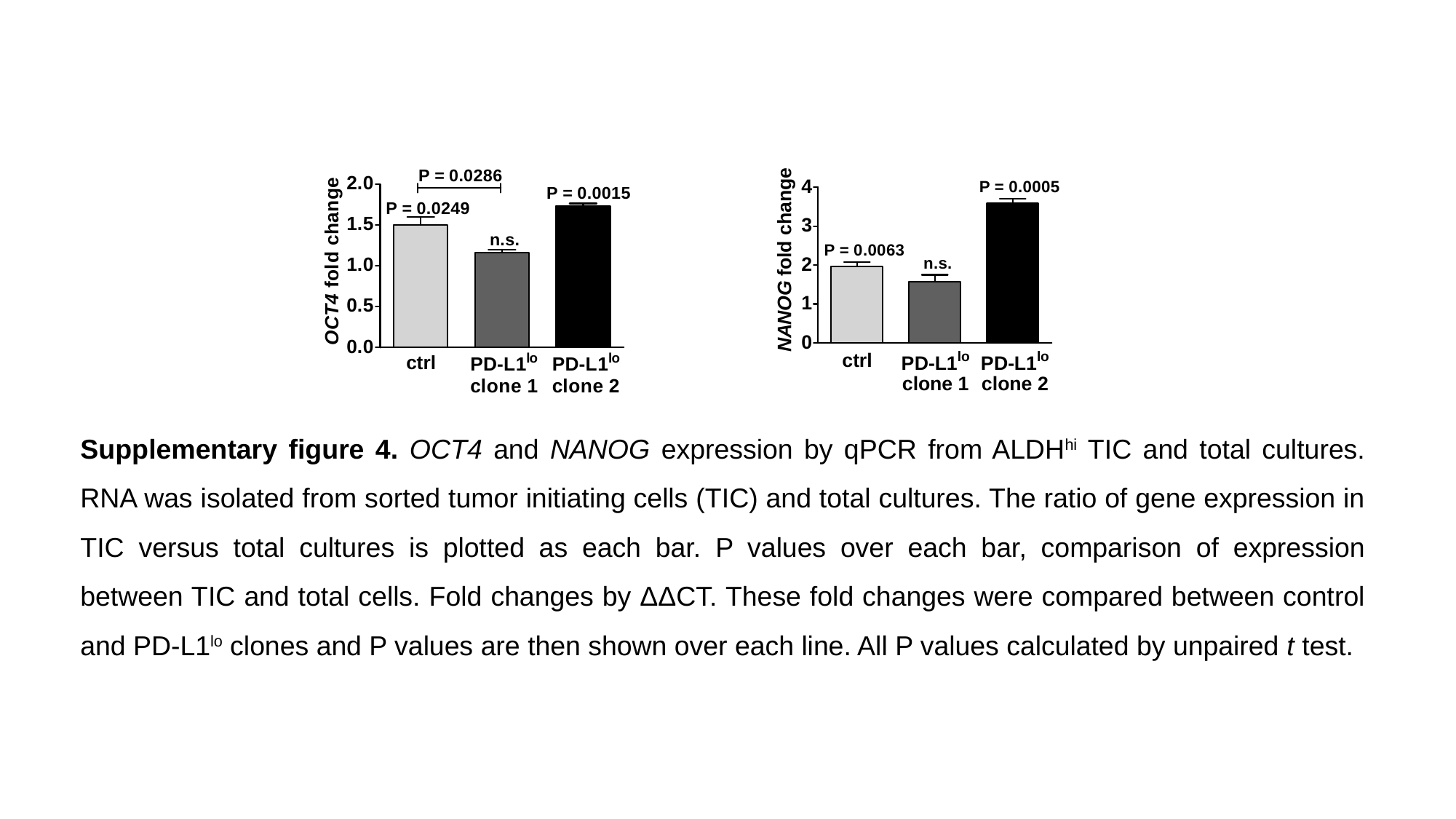

Supplementary figure 4. OCT4 and NANOG expression by qPCR from ALDHhi TIC and total cultures. RNA was isolated from sorted tumor initiating cells (TIC) and total cultures. The ratio of gene expression in TIC versus total cultures is plotted as each bar. P values over each bar, comparison of expression between TIC and total cells. Fold changes by ΔΔCT. These fold changes were compared between control and PD-L1lo clones and P values are then shown over each line. All P values calculated by unpaired t test.
